# Transfer-Film Stabilization by Reaction-Assisted GO/CNT/ZDMA/SiO_2_ Filler Networks in High-Temperature Rubber–Metal Sliding Contacts

**DOI:** 10.3390/polym18182254

**Published:** 2026-09-16

**Authors:** Deao Han, Xueru Zhong, Shuolei Wang, Yangkun Shao, Yongshuo Xing, Weihao Li, Zhanzhao Song, Feng Gong, Quanzhong Zhang, Chuansheng Wang, Deshang Han, Lin Wang

**Affiliations:** 1 College of Transportation, Ludong University, Yantai 264025, China; 2 Analysis and Testing Center, Ludong University, Yantai 264025, China; 3 Graduate School of Convergence Science and Technology, Seoul National University, Seoul 08826, Republic of Korea; 4 School of Mechatronics Engineering, Qingdao University of Science and Technology, Qingdao 266061, China

**Keywords:** rubber–metal sliding, transfer film, GO/CNT hybrid filler, zinc dimethacrylate, silica silanization, third-body wear

## Abstract

High-temperature rubber–metal sliding in internal mixers involves filler-network evolution, third-body generation and transfer-film formation. In this work, a reaction-assisted GO/CNT/ZDMA/SiO_2_ network was constructed through in situ formation of zinc dimethacrylate from ZnO/methacrylic acid, TESPT-mediated silanization of silica, and GO/CNT sheet-tube hybrid architecture. The key scientific contribution is the separation of mean friction from metal-wear suppression: the best wear resistance was obtained when the network maintained a continuous rubber/carbon-rich transfer film, not when the mean friction coefficient was lowest. At 3 phr GO with 3 phr CNT (C4), filler dispersion was more uniform, hard agglomerate release was suppressed, and metal wear volume reached 1.63 × 10^6^ μm^3^ with a volume-loss rate of 8.92%. Excessive GO promoted stacking and secondary agglomeration, increasing third-body supply, transfer-film rupture and metal exposure. Because no inert-atmosphere, pH, XPS, Raman, electrochemical or residue-concentration tests were performed, chemical assistance is treated as a possible secondary contribution after film rupture rather than as a proven independent mechanism. The results identify transfer-film continuity and friction fluctuation as practical screening indicators for reactive rubber–metal contacts.

## 1. Introduction

Internal mixers expose rotor end faces and chamber end faces to repeated rubber–metal contact under elevated temperature, strong shear and nonuniform contact stress. The wear of these metal components increases maintenance cost, contaminates compounds with metallic debris and shortens service life. Reducing metal damage therefore requires not only a low-friction rubber compound but also a composition that controls third-body generation and transfer-film continuity during high-temperature sliding [1,2,3,4,5,6].

Previous studies have shown that GO/CNT hybrid fillers can improve rubber reinforcement and tribological stability, that ZnO/MAA systems can generate in situ zinc dimethacrylate (ZDMA), and that silica-silane chemistry can improve filler–rubber compatibility [7,8,9,10,11,12,13,14,15,16,17,18,19,20,21,22,23,24,25,26,27,28,29,30,31]. Rubber wear and transfer-film formation are also governed by third-body mechanics, contact deformation and interfacial film evolution [4,5,6,32,33,34,35,36,37,38,39,40,41]. However, these lines of evidence do not yet clarify how a combined GO/CNT/ZDMA/SiO_2_ network regulates third-body release, transfer-film coverage and metal-counterface damage under a single elevated-temperature rubber–metal sliding condition.

In formulations containing ZnO/MAA-derived ZDMA and TESPT-silanized silica, the contact cannot be interpreted as a purely mechanical rubber–metal pair. Reactive reinforcing structures, carbon-based fillers, residual acidic species and ethanol–water vapor generated during silanization may coexist near the sliding interface. Nevertheless, direct chemical evidence for an independent corrosion or tribocorrosion mechanism is absent in the present data. The principal mechanism is therefore framed as transfer-film-controlled third-body wear, with possible chemical assistance only after transfer-film rupture and fresh-metal exposure [42,43,44,45,46,47,48,49,50].

This study evaluates a rubber composite system containing N330 carbon black, SiO_2_, in situ ZnO/MAA-derived ZDMA, TESPT, CNT and progressively increasing GO contents. The aim is to determine how the reaction-assisted filler network links filler dispersion, third-body supply, friction stability, transfer-film continuity and metal wear. We hypothesize that moderate GO loading improves the CNT-supported network and stabilizes the transfer film, whereas excessive GO promotes agglomeration, film rupture and severe metal exposure.

The scientific contribution is the evidence chain from filler-network structure to metal damage: a non-monotonic GO effect, the distinction between mean friction and wear suppression, and the identification of transfer-film continuity as the controlling criterion. This framework extends earlier filler/wear studies by connecting formulation-dependent dispersion with direct metal-counterface protection rather than evaluating rubber friction alone.

## 2. Experimental

### 2.1. Materials and Formulation Design

All raw materials were supplied by Yantai Taihong Rubber Co., Ltd. (Yantai, China) and used as received. TSR20 technically specified natural rubber, BR9000 high-cis polybutadiene rubber, and RS2557S solution-polymerized styrene–butadiene rubber were used as the rubber matrix. TSR20 had an initial plasticity (P_0_) of ≥30 and a plasticity retention index of ≥40, with dirt, ash, nitrogen, and volatile-matter contents of ≤0.16, ≤1.0, ≤0.60, and ≤0.80 wt%, respectively. BR9000 had a cis-1,4 content of approximately 94–96%, and its standard vulcanizate exhibited a 300% modulus of approximately 7.0–12.0 MPa. RS2557S provided balanced processability, wet-skid resistance, rolling resistance, and wear resistance. N330 furnace carbon black, with an average primary-particle size of approximately 30–35 nm, a nitrogen surface area of approximately 78 m^2^ g^−1^, and an oil absorption number of approximately 102 mL (100 g)^−1^; and 115MP precipitated silica, with a BET surface area of approximately 100–130 m^2^ g^−1^, were used as the principal reinforcing fillers. ZnO and methacrylic acid (MAA) were introduced to generate zinc dimethacrylate (ZDMA) in situ, while bis[3-(triethoxysilyl)propyl] tetrasulfide (TESPT) was used to promote silica silanization and rubber–silica interfacial coupling. CNTs and GO were employed as one-dimensional tubular and two-dimensional sheet-like nanofillers, respectively, to regulate the connectivity and stability of the hybrid filler network. The detailed formulations are presented in Table 1.

### 2.2. Instruments

The main instruments were an XSM-500 internal mixer (Shanghai Kechuang Rubber and Plastic Machinery Co., Ltd., Shanghai, China), a BL-6157 two-roll mill (Baolun Precision Testing Instrument Co., Ltd., Dongguan, China), a QLB-400 × 400 × 2 plate vulcanizer (Shanghai No. 1 Rubber Machinery Factory, Shanghai, China), a CSM tribometer (CSM Instruments SA, Peseux, Switzerland), a ZT-2588S steam generator (Zhiteng Co., Taiwan, China), a LEXT OLS5000 three-dimensional laser microscope (Olympus, Tokyo, Japan), an RPA2000 rubber process analyzer (Alpha Technologies, Akron, OH, USA), a DisperGRADER analyzer (Alpha Technologies, Akron, OH, USA) and a SU8000 field-emission scanning electron microscope (Hitachi Group, Tokyo, Japan). The tribological test was treated as a reproducible laboratory screening test rather than as a direct scale model of the spatially varying pressure and shear fields in an industrial mixer.

### 2.3. Sample Preparation

**Scheme 1:**
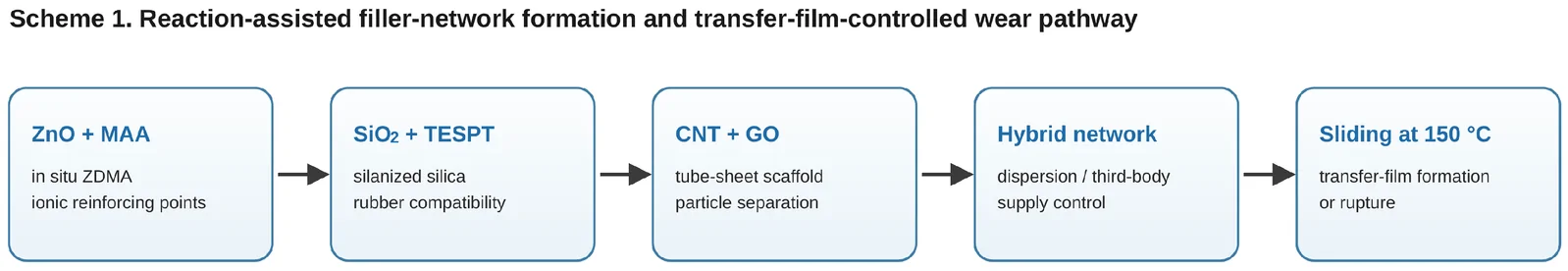
Reaction-assisted filler-network formation and transfer-film-controlled wear pathway. The scheme is based on the formulation and mechanistic interpretation in the manuscript and does not introduce new experimental data.

The rubber matrix was masticated on a two-roll mill, cut into narrow strips and then fed into the internal mixer. Mixing was conducted at 70 r/min with a fill factor of 0.65. The rubber matrix, chemicals and fillers were added sequentially, and the final discharge temperature was approximately 150 °C. The compound was subsequently blended with the vulcanization/crosslinking system on the two-roll mill and sheeted for further testing. Scheme 1 summarizes the intended formation route from ZnO/MAA to ZDMA, TESPT/SiO_2_ silanization, CNT/GO scaffold formation and transfer-film-controlled sliding response.

Two reaction stages are central to the formulation. First, MAA reacts with ZnO in the low-to-medium temperature range to form ZDMA, which provides ionic reinforcing points and salt-bonded crosslinking structures. Second, TESPT hydrolyzes and condenses with silica surface silanol groups during heating, improving silica-rubber compatibility and reducing filler polarity. Because mixing and sliding were conducted under relatively sealed and high-temperature conditions, residual acidic MAA and ethanol–water vapor were considered as possible contributors to mechanically triggered chemical exposure after transfer-film rupture.

This interpretation is used cautiously: the present work does not claim independent corrosion control but treats the acidic/polar environment as a possible amplifier of wear after fresh-metal exposure.

### 2.4. Characterization and Testing

RPA strain sweeps were conducted at 60 °C and 0.01 Hz over 0.28–40% strain. The Payne effect was expressed as ΔG′, defined as the difference between low-strain and high-strain storage modulus, to evaluate filler-network strength and breakdown.

For SEM observation, rubber specimens were fractured after liquid-nitrogen treatment, and worn rubber and metal surfaces were observed using field-emission SEM. Representative worn metal regions were analyzed by EDS to identify metal exposure and transfer-film-related elements. Filler dispersion was evaluated using a DisperGRADER analyzer.

Friction and wear tests were performed using a CSM tribometer at 150 °C for 60 min under a load of 5 N and a rotational speed of 70 r/min. These conditions were selected as reproducible laboratory screening conditions that generated measurable friction and wear without catastrophic specimen damage; they are not intended as a direct scale model of the nonuniform pressure and shear fields in an industrial mixer. The metal counterpart was made of the same material as the internal-mixer end face. The mean coefficient of friction, standard deviation and coefficient of variation during the stable stage were used to describe interfacial shear resistance and stability. Wear volume, volume-loss rate and surface roughness were obtained using the LEXT OLS5000 three-dimensional laser microscope. Unless raw replicate datasets are supplied, these values are presented as descriptive measurements rather than inferential statistics. The test plan is summarized in Table 2.

## 3. Results and Discussion

### 3.1. Construction of Reaction-Assisted GO/CNT/ZDMA/SiO_2_ Multiphase Filler Networks

The filler network was designed through three coupled structural units: in situ ZDMA ionic reinforcement, TESPT-silanized SiO_2_ and a GO/CNT sheet-tube hybrid framework. ZDMA provides hard ionic domains, silanized silica improves filler–rubber compatibility, and CNT/GO regulates the spatial distribution of reactive particles. The key question is whether these units form a uniform network that supports a stable transfer film, or whether they become local agglomerates that supply hard third-body particles.

C1 contained no CNT/GO hybrid structure; therefore, SiO_2_, N330 carbon black and in situ ZDMA were dispersed mainly through mechanical mixing and local chemical interactions. In this case, large reinforcing regions could detach under high-temperature shear and damage the metal as third bodies. After CNT and GO were introduced, the tubular CNT skeleton and sheet-like GO surfaces improved particle adsorption, bridging and separation. At an appropriate GO loading, this structure helped distribute silanized SiO_2_ and ZDMA more uniformly and reduced local stress concentration.

Excessive GO, however, may reverse this beneficial effect. Sheet stacking and GO/CNT entanglement increase filler–filler interactions, promote hard agglomerates and interrupt the regeneration of a continuous transfer film. The following sections therefore focus on the relationship among network strength, dispersion, third-body release, transfer-film stability and metal wear.

### 3.2. Filler-Network Structure and Payne Effect

**Figure 1:**
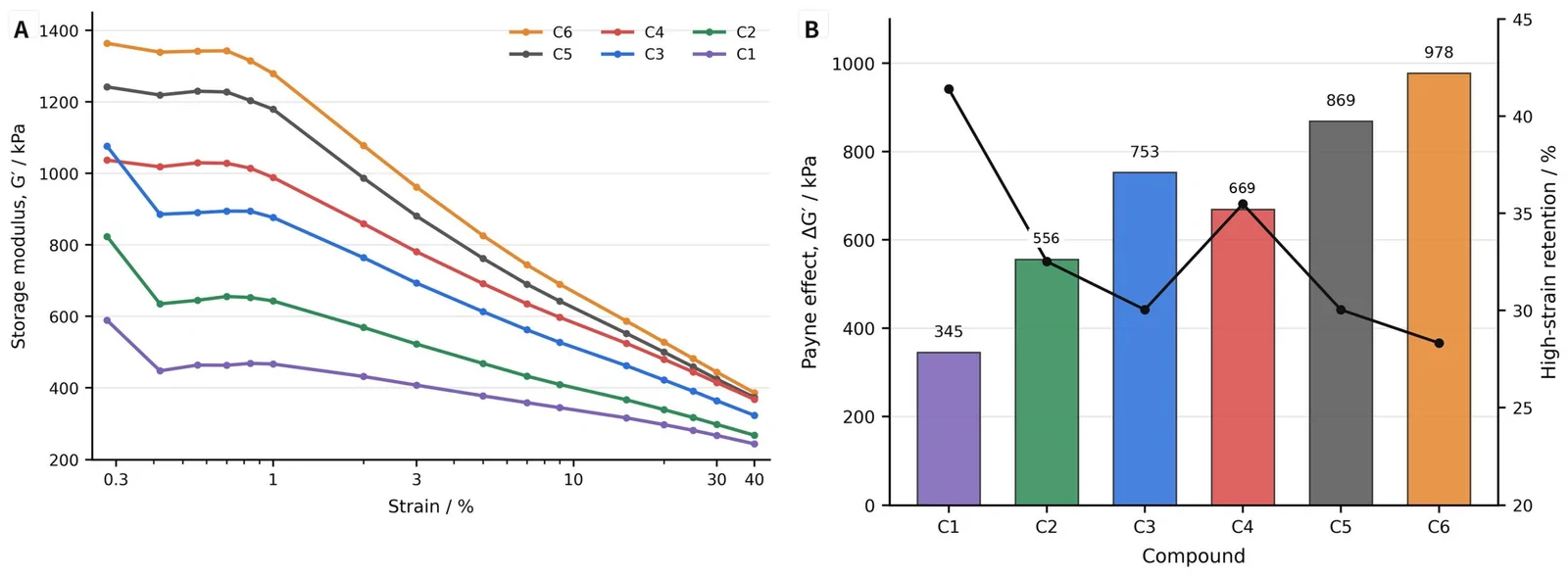
Strain-sweep response and Payne-effect analysis of C1–C6 rubber composites. (**A**) Storage modulus G′ as a function of strain, showing progressive filler-network breakdown under increasing deformation. (**B**) Payne effect ΔG′ of different formulations, used to evaluate filler-network strength and homogeneity. Bars show ΔG′, and the black line shows high-strain retention (%) on the right axis. C1 is the CNT/GO-free reference; C2–C6 contain 3 phr CNT and 1–5 phr GO, respectively.

All formulations showed a decrease in storage modulus G′ with increasing strain, consistent with the progressive breakdown of the filler network under large-amplitude shear. The Payne effect in this system reflects both physical filler–filler interactions among carbon black, SiO_2_, CNT and GO and reaction-assisted interactions associated with ZDMA and TESPT-silanized silica (Figure 1).

The ΔG′ value decreased from 752.83 kPa for C3 to 669.04 kPa for C4, indicating that 3 phr GO produced a more balanced network with sufficient reinforcement but fewer large agglomerates. By contrast, ΔG′ increased to 868.91 and 977.55 kPa for C5 and C6, respectively, suggesting stronger filler–filler interactions and GO stacking at excessive GO loadings. Thus, C4 represents the most favorable compromise between network strength and network homogeneity.

A stronger Payne effect is therefore not necessarily beneficial. In this tribological system, excessive network strength can indicate agglomeration and potential third-body sources. The moderate ΔG′ of C4 suggests a lower tendency for hard-particle release and a higher potential for transfer-film protection.

### 3.3. Filler Dispersion and Its Contribution to Third-Body Wear

**Figure 2:**
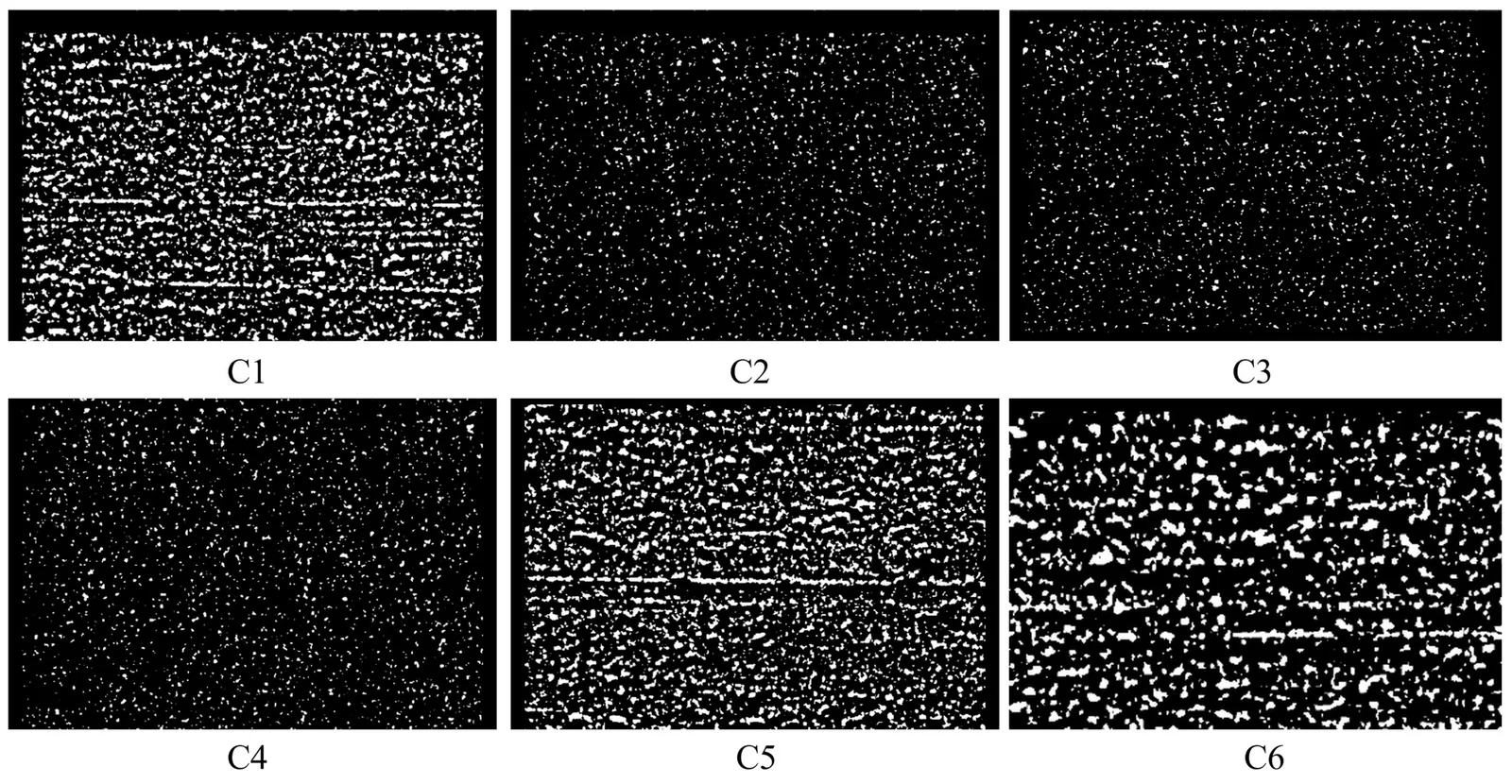
Filler-dispersion morphology of C1–C6 rubber composites. The panels show the decrease in visible agglomerates at moderate GO loading and re-agglomeration at excessive GO loading. C1 is the CNT/GO-free reference; C2–C6 contain 3 phr CNT and 1–5 phr GO, respectively.

The dispersion images are consistent with the RPA results. C1 retained obvious agglomerated regions because no GO/CNT hybrid framework was present. From C2 to C4, the agglomerates decreased, the particle size became smaller and the distribution became more uniform, showing that moderate GO supported by CNT could separate silanized SiO2, carbon black and in situ ZDMA particles (Figure 2).

Agglomeration increased again in C5 and C6, indicating that excessive GO caused sheet stacking and secondary aggregation. These agglomerates can be detached during sliding and converted into third-body abrasives. They also create weak interfacial regions in the rubber surface, accelerating crack initiation and debris release.

Thus, filler dispersion is the bridge between formulation chemistry and metal wear. Improved dispersion favors compact transfer-film formation and limits fresh-metal exposure, whereas poor dispersion increases film defects and allows hard particles and reactive small molecules to reach the real contact area.

### 3.4. Coefficient of Friction and Interfacial Stability

**Figure 3:**
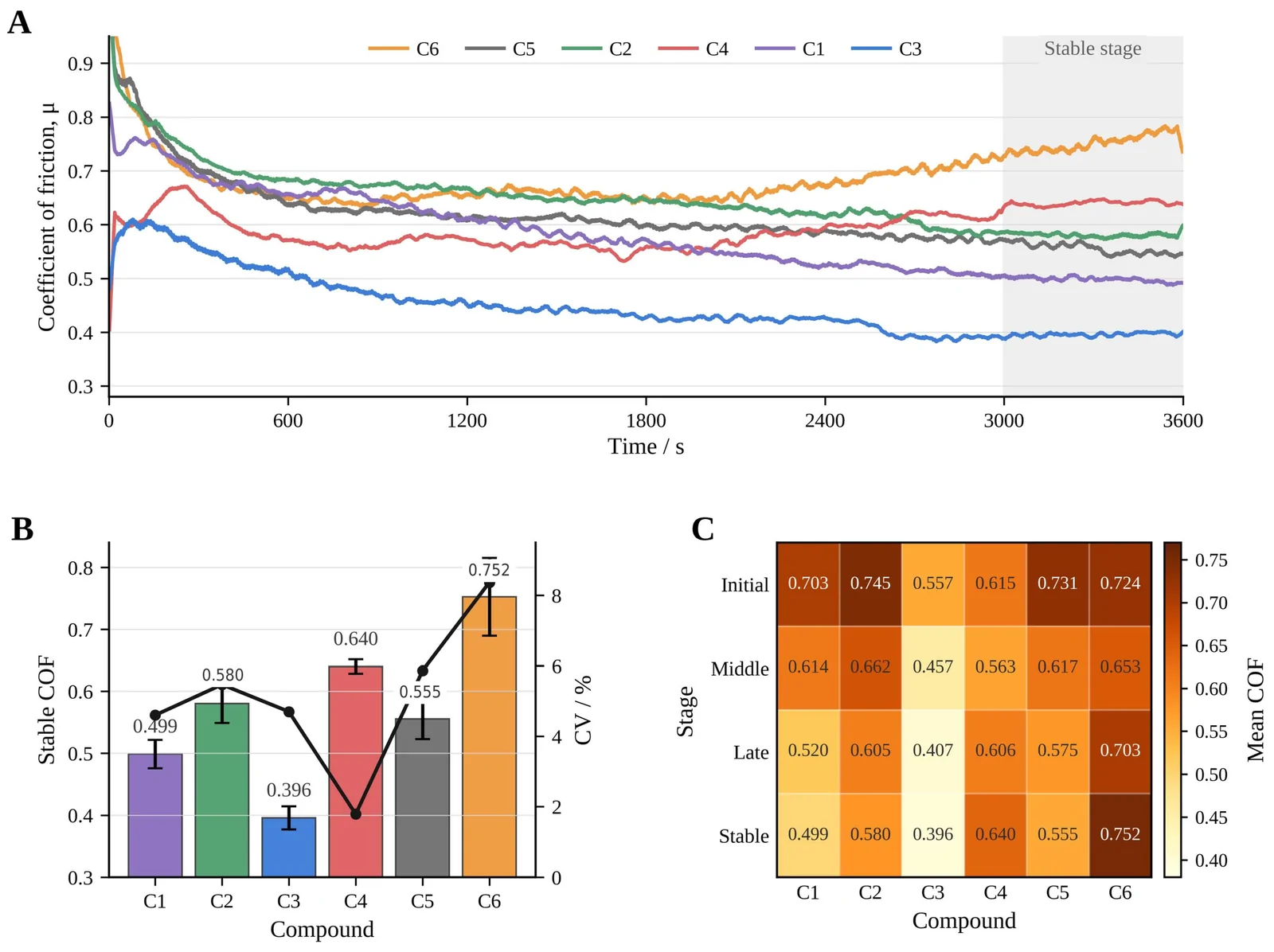
Coefficient of friction and interfacial stability of C1–C6 rubber composites. (**A**) Time-dependent coefficient of friction during 3600 s sliding at 150 °C and 5 N. (**B**) Stable-stage mean friction coefficient with standard-deviation error bars and the corresponding coefficient of variation; the black line shows the coefficient of variation on the right axis. (**C**) Stage-resolved heat map of mean friction coefficient in the initial, middle, late and stable stages. C4 shows the lowest fluctuation, whereas C6 shows a sustained increase and larger fluctuations.

The coefficient of friction reflects the dynamic formation, rupture and regeneration of the transfer film. C3 exhibited the lowest stable-stage mean friction coefficient, whereas C4 showed the smallest fluctuation and coefficient of variation. For C4, the mild time-dependent increase is interpreted as a running-in response associated with progressive real-contact-area development and compaction of a comparatively continuous transfer film; its low fluctuation indicates a stable interface. For C6, the sustained increase and larger fluctuations are consistent with intermittent detachment of GO-rich agglomerates, repeated transfer-film rupture and enhanced third-body plowing (Figure 3).

All RPA and tribological tests were repeated five times (*n* = 5) on independent specimens. Results are reported as mean ± standard deviation (SD); variance (SD^2^) and the coefficient of variation (CV = SD/mean × 100%) are also provided. For the stable-stage coefficient of friction, the per-compound statistics were: C1 0.499 ± 0.023, C2 0.580 ± 0.031, C3 0.396 ± 0.019, C4 0.640 ± 0.012, C5 0.555 ± 0.033 and C6 0.752 ± 0.062 (CV 4.6%, 5.3%, 4.7%, 1.8%, 5.9% and 8.3%, respectively).

These results show that a low mean coefficient of friction is not equivalent to low metal wear. In C4, the small fluctuation indicates reduced third-body supply and a transfer film that remained comparatively continuous under shear. Because only 150 °C was tested, the present data do not establish a quantitative temperature dependence of friction or wear; systematic multi-temperature testing is required to separate thermal softening, transfer-film compaction and possible chemical assistance.

Therefore, the stable-stage standard deviation and coefficient of variation are useful indicators of transfer-film reliability. Large fluctuations, as observed for C5 and C6, imply repeated film rupture, metal exposure and removal of weak interfacial products under shear.

### 3.5. Metal Wear Volume and Surface Roughness Change

**Figure 4:**
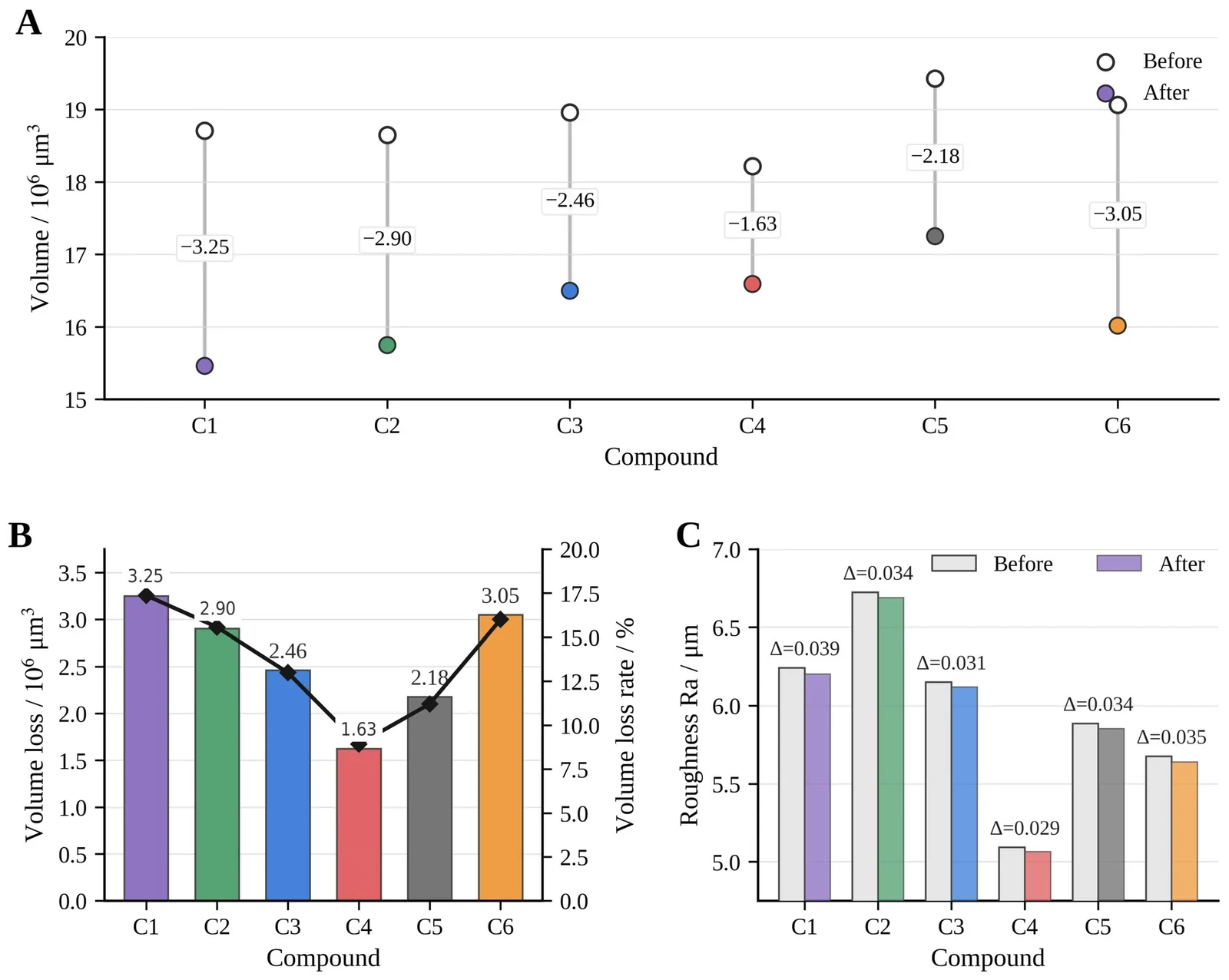
Metal wear volume, volume-loss rate and surface roughness before and after sliding for C1–C6. (**A**) Metal-surface volume before and after sliding, with the marked volume-change values. (**B**) Volume loss and volume-loss rate of the metal counterpart. (**C**) Surface roughness Ra before and after sliding. Colors identify C1–C6 consistently across the panels; in (**A**,**C**), open/white and filled/colored markers or bars indicate the values before and after sliding, respectively. C4 shows the lowest wear volume and smallest roughness change.

Metal wear volume provides direct evidence for the protective effect of the optimized network. The volume change first decreased and then increased with increasing GO content. C4 had the lowest wear volume, 1.63 × 10^6^ μm^3^, and the lowest volume-loss rate, 8.92%. When the GO loading increased further, the wear volume increased again, and C6 approached the high wear level of C1 (Figure 4).

The roughness change followed the same trend. C4 showed the smallest Ra change, indicating the lowest surface-relief variation. These results show that wear reduction was achieved not by the lowest shear resistance but by improved reactive-filler dispersion, reduced third-body particle supply and a stable transfer film.

The increase in wear for C5 and C6 shows that the GO/CNT effect has an optimum range. Excessive GO produces agglomerates that raise local contact pressure, cut the transfer film and generate deeper grooves. Once grooves and peeling pits form, they may retain acidic or polar species, but the present data do not quantify surface chemistry or establish an independent chemical-wear mechanism.

### 3.6. SEM/EDS Analysis of Worn Metal Surfaces

SEM observations further support this mechanism. The C1 surface showed grooves, scratches and local peeling, indicating simultaneous plowing and transfer-film rupture. In C2 and C3, the number of grooves decreased and film-like coverage became more evident, suggesting that the CNT/GO hybrid network began to promote transfer-film formation.

The C4 metal surface was comparatively smooth and covered by a more continuous gray film. Deep grooves and large peeling areas were strongly suppressed. This morphology indicates that the transfer film isolated the metal substrate, buffered local shear and reduced direct contact with hard particles. In C5 and C6, attached particles, deeper grooves and film rupture reappeared, demonstrating the harmful effect of excessive GO-induced agglomerates.

**Figure 5:**
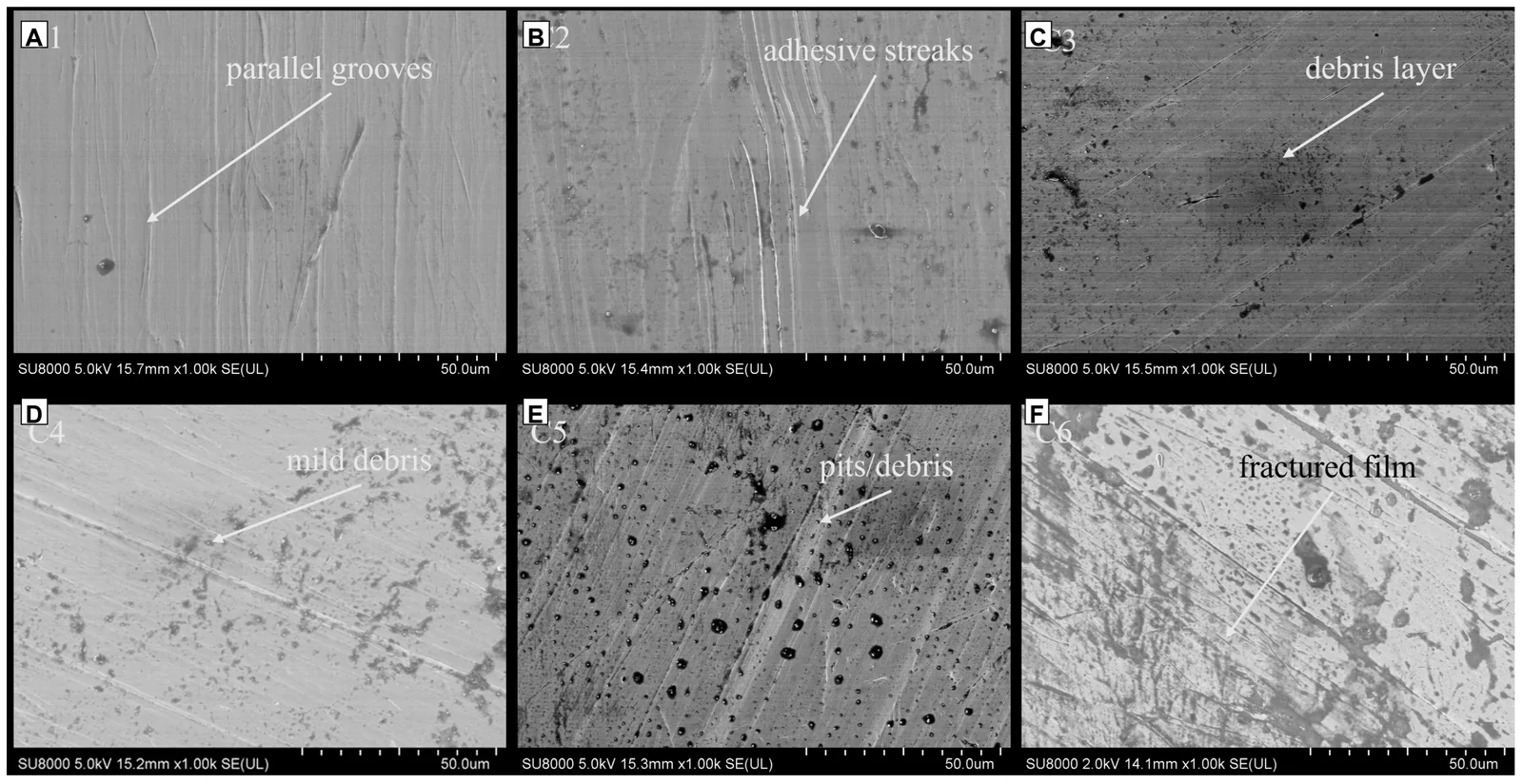

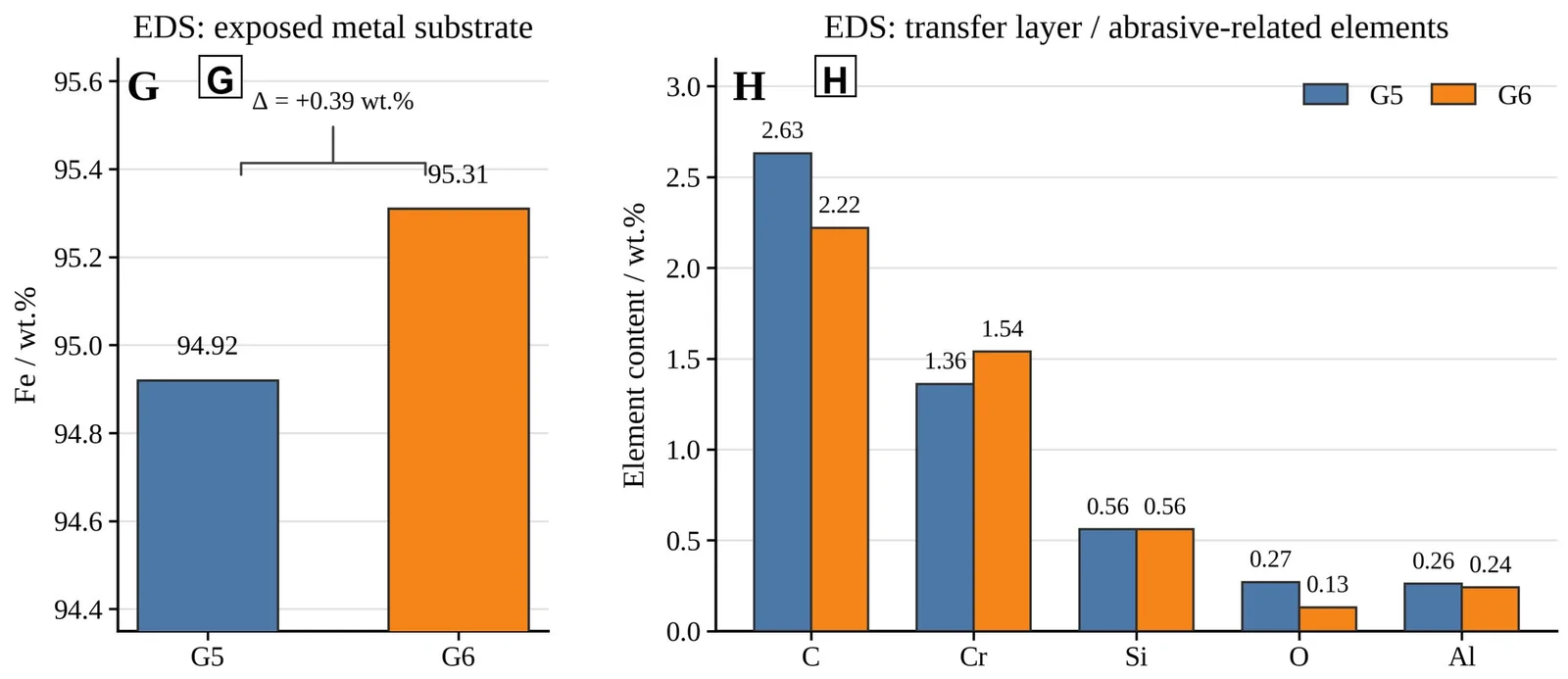
SEM morphologies and representative EDS results of worn metal surfaces after sliding. (**A**) C1, parallel grooves; (**B**) C2, adhesive streaks; (**C**) C3, debris layer; (**D**) C4, mild debris and a comparatively film-covered surface; (**E**) C5, pits and debris; (**F**) C6, fractured film and severe surface damage; (**G**) Fe content comparison between C5 and C6; and (**H**) C, Cr, Si, O and Al contents from representative EDS analysis. Moderate GO promotes smoother film-covered surfaces, whereas excessive GO leads to grooves, exposed metal and third-body abrasion.

EDS supports this interpretation. Compared with C5, C6 showed higher Fe content and lower C and O contents, indicating stronger metal exposure and weaker rubber/carbon-rich film coverage. The presence of Si suggests that silica-containing debris participated in third-body abrasion. The low O content does not exclude mechanically triggered chemical effects because weak oxygen-containing products may be continuously removed under high shear (Figure 5).

Accordingly, the EDS results are interpreted as final-surface evidence after repeated exposure and removal, rather than as direct proof of a stable oxide layer, corrosion process or independently established mechano-chemical reaction. No inert-atmosphere, pH, surface-residue, XPS, Raman or electrochemical measurements were performed.

### 3.7. SEM Observations of Worn Rubber Surfaces and Sources of Third-Body Particles

The worn rubber surfaces show that the third-body source originates from the rubber side. Particle adhesion, compacted debris, crack growth, local tearing and blocky peeling occurred simultaneously. Bright particles and irregular agglomerates indicate that filler particles, rubber fragments and possible metal debris jointly participated in the interfacial third-body layer.

**Figure 6:**
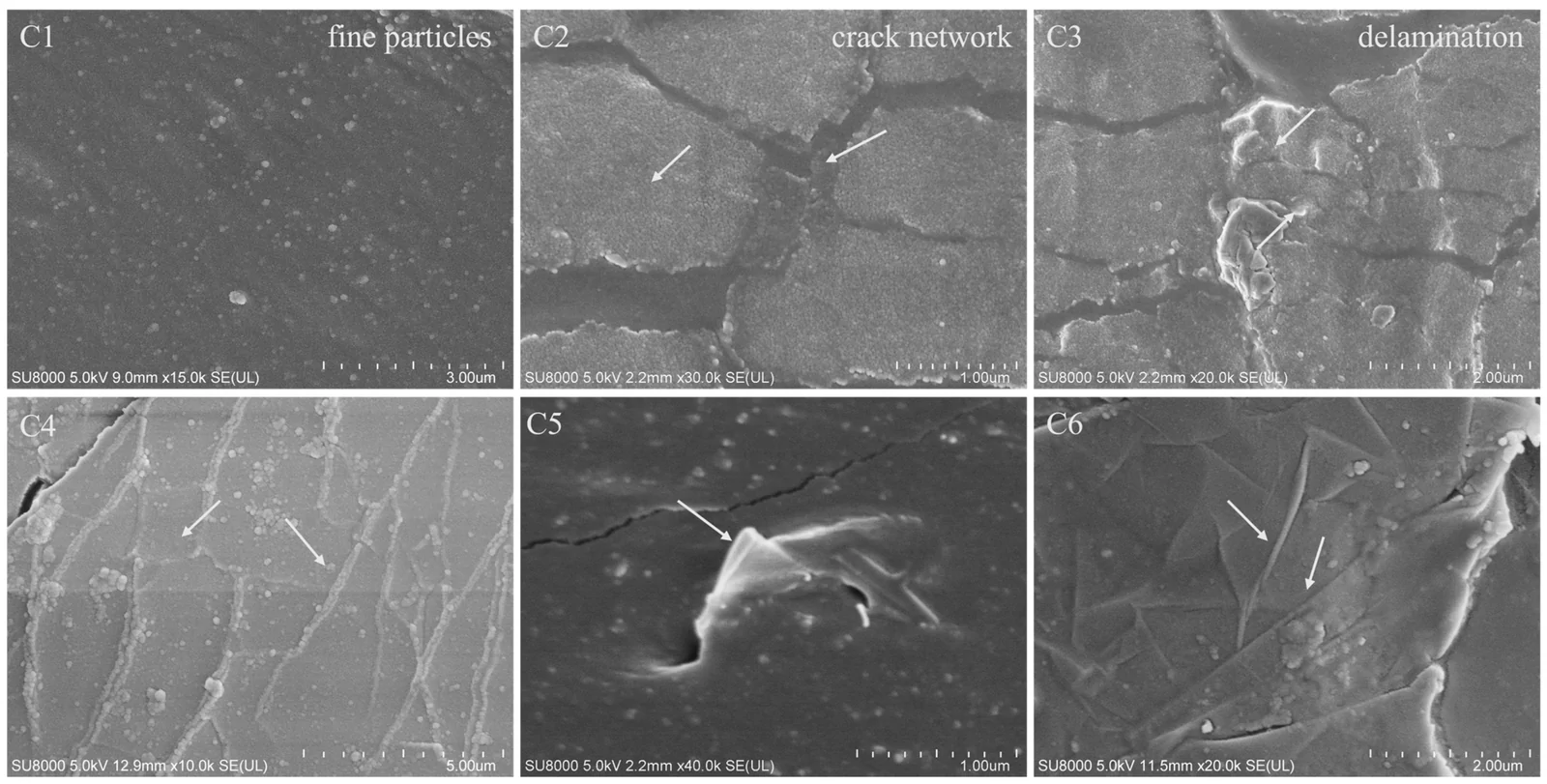
Representative SEM morphologies of worn rubber surfaces for C1–C6. C1 is the CNT/GO-free reference; C2–C6 contain 3 phr CNT and 1, 2, 3, 4 and 5 phr GO, respectively. The images show the evolution of fine particles, crack networks, delamination, adhesion, tearing and lamellar peeling on the worn rubber surfaces. Arrows identify representative cracks, delamination, adhered particles or debris and lamellar tearing.

Local filler agglomeration and inhomogeneous reactive networks created stress concentration in the worn rubber surface. When agglomerates of silanized SiO_2_, in situ ZDMA, carbon black or GO/CNT were insufficiently bonded, interfacial debonding and local tearing occurred under high-temperature shear. Detached particles could either be compacted into a temporary protective layer or become free abrasives after film rupture (Figure 6).

**Figure 7:**
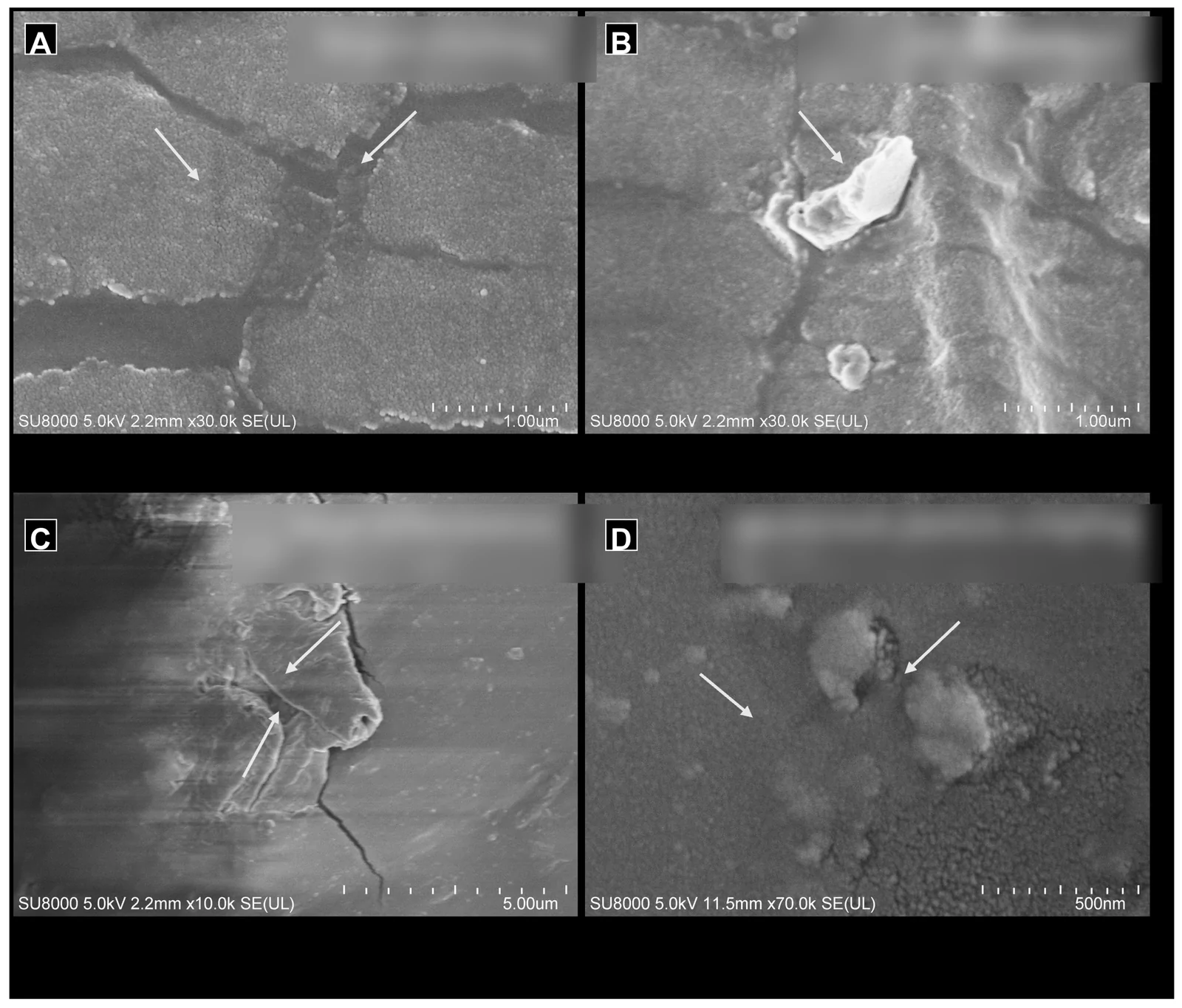
SEM details of rubber wear mechanisms. (**A**) Fatigue crack propagation; (**B**) hard particles/agglomerates; (**C**) local delamination and debonding pit; and (**D**) microcrack-particle coupling. Arrows indicate crack paths, hard particles or agglomerates, delamination or debonding and microcrack–particle coupling. These features represent possible sources and transport pathways of third-body particles during rubber–metal sliding.

Cracking and peeling of the rubber surface influence metal wear by controlling the supply of mixed third bodies. Compact debris may temporarily protect the metal, but large or poorly adhered particles become cutting abrasives. Cracks and peeling boundaries can also provide pathways for residual acidic MAA and ethanol–water vapor to enter the real contact zone after film rupture (Figure 7).

### 3.8. Three-Dimensional Morphological Evolution of Metal and Rubber Surfaces

Three-dimensional morphology provides height-direction evidence for transfer-film stability. Before sliding, the metal surface mainly showed machining scratches. Mildly worn regions showed shallow grooves and film-like coverage with limited height variation, indicating that a stable transfer film constrained the metal relief and reduced direct exposure of the substrate.

**Figure 8:**
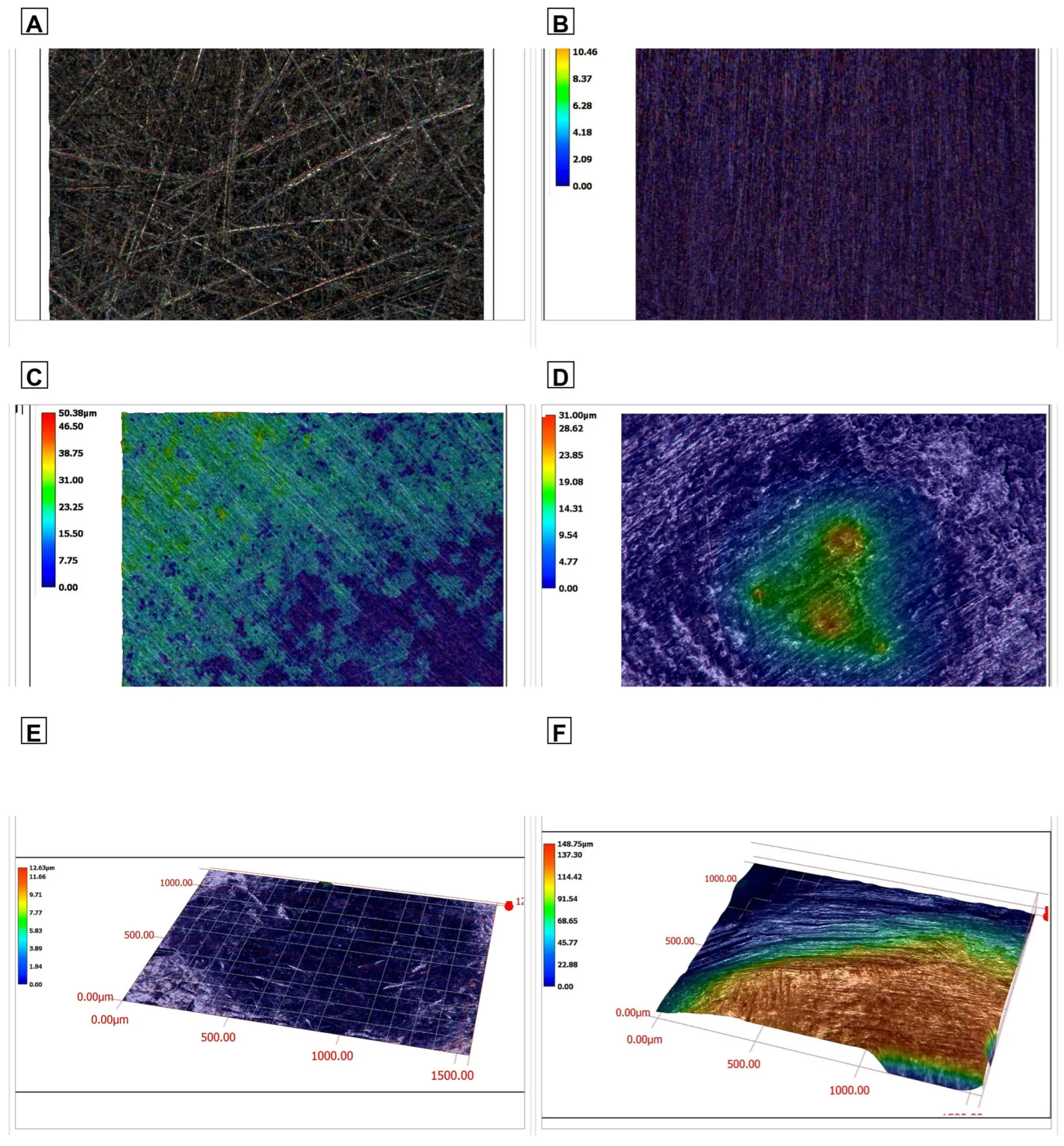
Three-dimensional morphological evolution of metal surfaces before and after sliding. (**A**) Reference scratches before sliding; (**B**) shallow grooves after mild wear; (**C**) transfer-film-covered region; (**D**) circular texture/local height variation; (**E**) stable-interface 3D profile; and (**F**) pile-up/peeling 3D profile after severe wear. Mild wear is associated with shallow grooves and film coverage, whereas severe wear shows pile-up, peeling and large height variation.

Severely worn regions showed a much larger height range, pronounced grooves, ridge-like pile-up and local peeling. The rubber surface also showed large protrusions, pits and tearing boundaries. These features indicate repeated cycles of agglomerate detachment, debris accumulation, transfer-film formation and film rupture. Local height gradients therefore reflect mechanical plowing and possible entry sites for reactive media after the protective film fails, but they do not quantify film thickness or coating quality (Figure 8).

### 3.9. Wear Mechanism Governed by Transfer-Film Stability in the Reaction-Assisted Filler Network

The combined results support a transfer-film-controlled wear mechanism. The beneficial pathway occurs when in situ ZDMA, TESPT-silanized SiO_2_ and the GO/CNT hybrid network form a uniform reactive filler structure. In this pathway, fewer hard agglomerates are released, rubber/carbon-rich debris spreads into a continuous transfer film, and the metal substrate is isolated from direct plowing and chemical exposure. C4 follows this pathway.

The failure pathway is dominated by excessive GO-induced agglomeration. Sheet stacking and GO/CNT entanglement transform the network into a heterogeneous hard-particle source. The released third bodies cut the transfer film and expose the metal substrate, producing deep grooves, pile-up and peeling. C6 follows this pathway, as shown by its high friction fluctuation, high Fe exposure and severe three-dimensional height variation.

A possible chemical contribution may become relevant only after the transfer film is damaged. Residual acidic MAA and ethanol–water vapor may contact fresh metal through cracks, grooves and peeling pits; promote weak interfacial products or reduce film adhesion; and then be removed by continued shear. This contribution remains a hypothesis because direct chemical verification was not performed.

**Figure 9:**
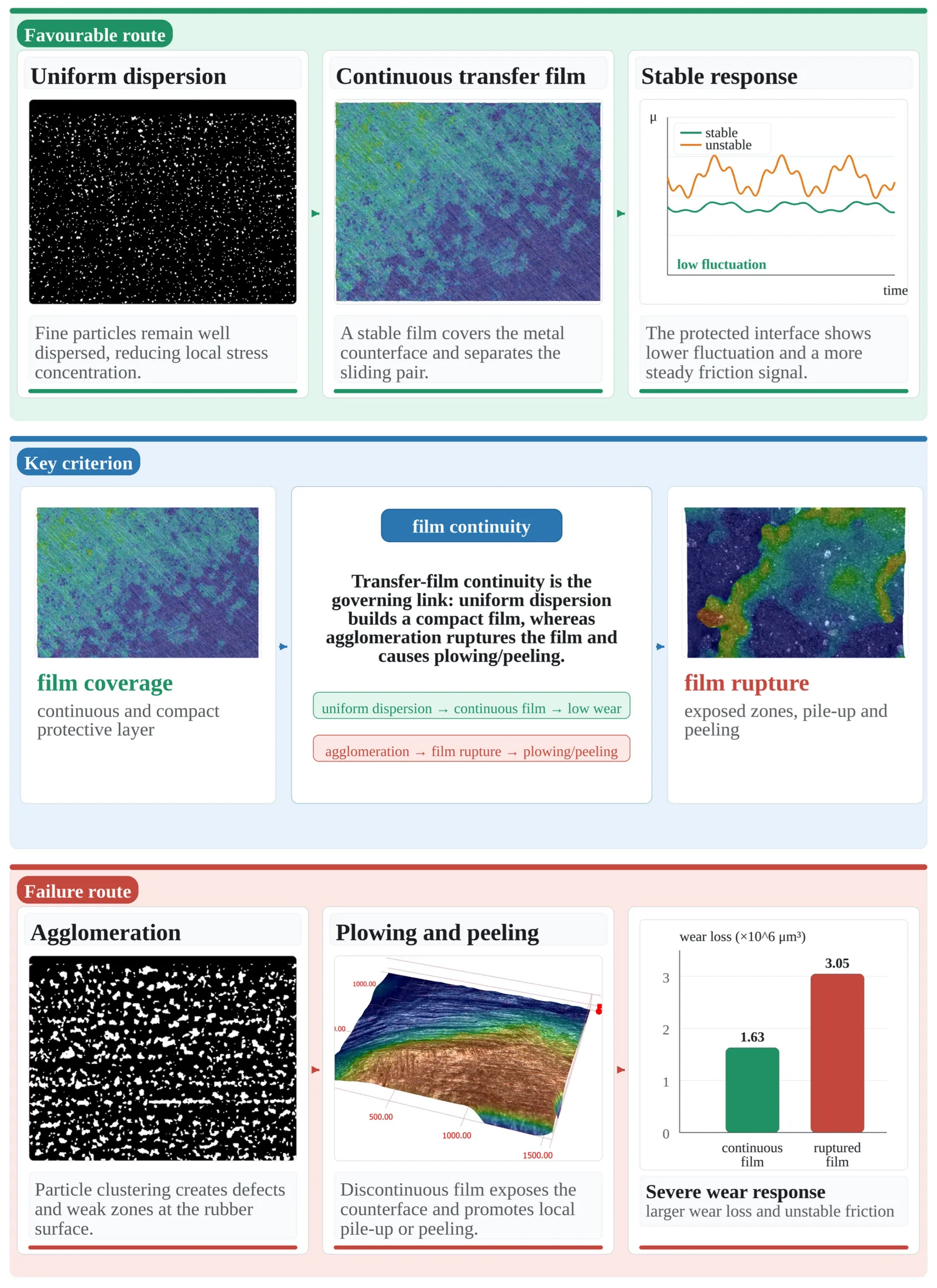
Correlation mechanism among filler dispersion, transfer-film coverage, friction stability and severe wear response. Uniform dispersion favors a stable transfer film, whereas agglomeration produces film rupture and high wear. Green denotes the favorable stabilization pathway, blue denotes the key transfer-film criterion and red denotes the failure pathway. The scheme summarizes the evidence chain from friction, SEM/EDS and three-dimensional morphology.

**Figure 10:**
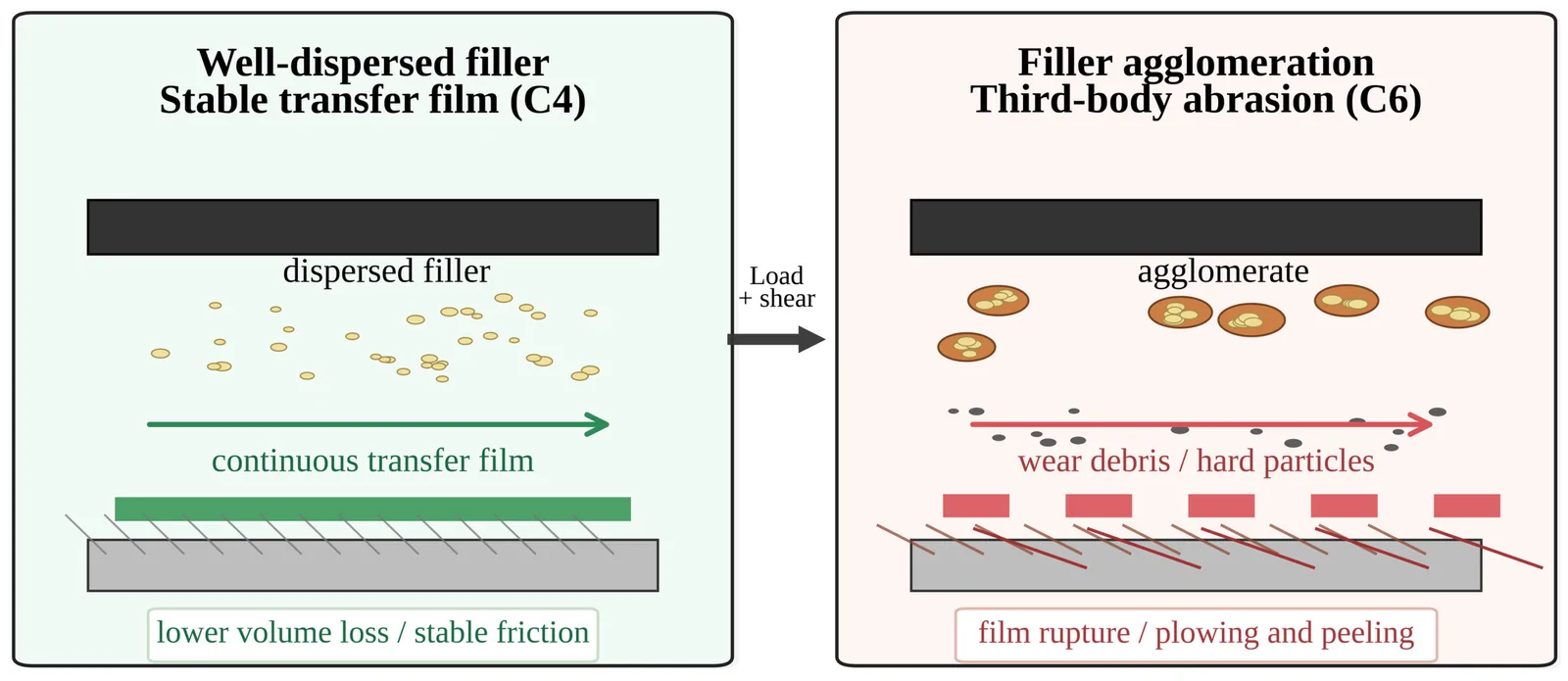
Schematic illustration of transfer-film stabilization and third-body abrasion in the reaction-assisted rubber–metal contact. The possible chemical-assistance pathway is shown as conditional on transfer-film rupture and fresh-metal exposure.

**Figure 11:**
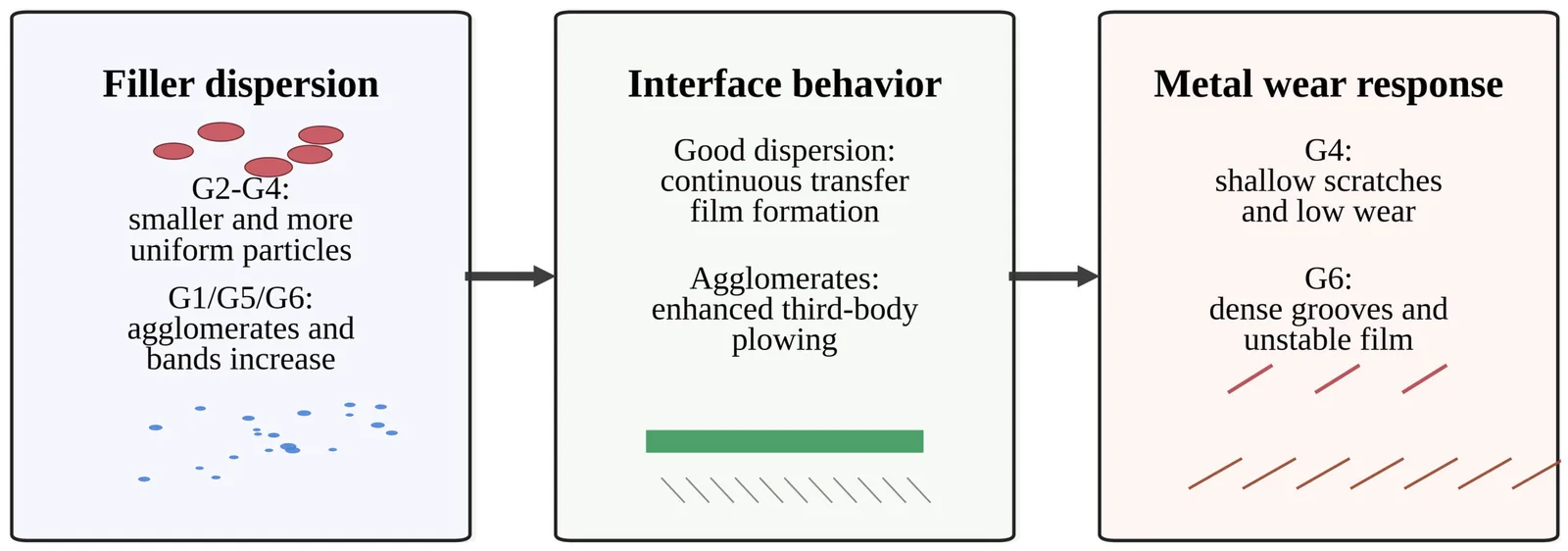
Correlation among filler dispersion, interface behavior and metal wear response, emphasizing the transition from stable transfer-film formation to plowing and unstable film failure. The figure summarizes qualitative mechanism evidence from friction, SEM/EDS and 3D morphology.

Figure 9, Figure 10 and Figure 11 summarize the evidence chain: filler dispersion controls third-body supply; third-body supply controls transfer-film continuity; transfer-film continuity determines metal exposure; and metal exposure controls the probability of mechanically triggered chemical assistance. Thus, the optimum formulation is the one that maintains a stable transfer film, not necessarily the one with the lowest mean friction coefficient.

### 3.10. Evidence Boundary: Transfer-Film-Controlled Third-Body Wear and Possible Chemical Assistance

The roles of MAA, high-temperature ethanol and water vapor are understood within a clear evidence boundary. MAA is essential for ZDMA formation, and TESPT silanization can generate ethanol during hydrolysis and condensation. Under high-temperature shear, acidic or polar species may assist local surface activation after the transfer film is broken. However, the present study did not directly measure pH, surface speciation, oxide thickness or electrochemical response; therefore, chemical assistance is treated as a conditional contribution rather than a dominant independent mechanism.

For C4, improved dispersion and reduced hard-particle release maintained continuous film coverage and limited fresh-metal exposure. For C5 and C6, agglomeration created repeated film rupture, grooves and active metal sites, allowing acidic and polar species to participate in mechanically triggered surface activation and shear removal.

Therefore, the present mechanism is best described as transfer-film-controlled third-body wear with possible chemical assistance. Further inert-atmosphere tests, pH or residue analysis, XPS, Raman mapping, electrochemical testing, EDS mapping or in situ tribological analysis would be required to evaluate transfer-film chemistry and metal oxidation states directly. This limitation is stated explicitly to avoid overinterpreting the EDS and morphology results.

Compared with prior work on GO/CNT rubber hybrids, ZDMA-reinforced rubber, silica-silane reinforcement, rubber wear and transfer-film/third-body tribology [1,2,3,4,5,6,7,8,9,10,11,12,13,14,15,16,17,18,19,20,21,22,23,24,25,26,27,28,29,30,31,32,33,34,35,36,37,38,39,40,41], this study focuses on how the combined filler network affects the metal counterpart. The present results fit those studies by showing that filler dispersion and interfacial film stability are linked but extend them by separating mean friction from metal wear and by connecting GO overloading to transfer-film rupture and metal exposure.

For the present formulation and test condition, 3 phr GO combined with 3 phr CNT provided the best balance between network homogeneity, friction stability and metal-wear suppression. Higher GO loadings should be avoided unless additional dispersion control is introduced. Friction fluctuation, coefficient of variation and transfer-film continuity are more useful screening indicators than the mean friction coefficient alone, although industrial validation under real mixer contact conditions is still required.

## 4. Conclusions

This study shows that metal wear in high-temperature reactive rubber–metal sliding contacts is governed primarily by transfer-film stability rather than by the mean coefficient of friction alone. A reaction-assisted GO/CNT/ZDMA/SiO_2_ network was constructed through ZnO/MAA in situ ZDMA formation, TESPT-mediated silica silanization and GO/CNT sheet-tube hybridization. At moderate GO loading (C4), reactive fillers were more uniformly dispersed, hard agglomerate release was reduced, and a continuous transfer film protected the metal counterpart. Excessive GO disrupted this pathway by promoting agglomeration, third-body plowing, transfer-film rupture and metal exposure. The mechanism is therefore assigned to transfer-film-controlled third-body wear with possible chemical assistance only after film rupture. For composition screening, 3 phr GO with 3 phr CNT is recommended under the present 150 °C/5 N laboratory condition, while multi-temperature testing, statistical analyses of replicate data and direct chemical-surface analyses are needed before broader industrial claims are made.

## Figures and Tables

**Table 1:** Formulations of the rubber composites (phr).

Ingredient (phr)	C1	C2	C3	C4	C5	C6
TSR20	30	30	30	30	30	30
BR9000	30	30	30	30	30	30
RS2557S	40	40	40	40	40	40
N330	10	10	10	10	10	10
SiO_2_	30	30	30	30	30	30
MAA	15	15	15	15	15	15
ZnO	7.5	7.5	7.5	7.5	7.5	7.5
CNT	0	3	3	3	3	3
GO	0	1	2	3	4	5
DCP	1.5	1.5	1.5	1.5	1.5	1.5
SAD	2	2	2	2	2	2
Si69	3	3	3	3	3	3
DPG	1.3	1.3	1.3	1.3	1.3	1.3

Note: C1 is the CNT/GO-free reference formulation; C2–C6 contain 3 phr CNT and 1, 2, 3, 4 and 5 phr GO, respectively.

**Table 2:** Test Plan.

Step	Purpose	Current Manuscript Basis	Remark
Formulation and mixing	Prepare C1–C6 GO-loading series	Table 1 and Section 2.3	Material details are described in Section 2.1.
RPA and dispersion	Assess filler-network strength and dispersion	Section 2.4, Section 3.2 and Section 3.3	Quantitative tests were repeated five times.
Tribology	Measure friction stability and metal wear	Section 2.4 and Figures 3 and 4	Quantitative tests were repeated five times.
SEM/EDS and 3D morphology	Assess transfer-film continuity and wear traces	Section 3.6, Section 3.7 and Section 3.8	Panel mapping and scale bars are described in the figure captions.

## Data Availability

The data presented in this study are available on reasonable request from the corresponding author. The data are not publicly available at this stage because they include raw experimental records, formulation-related datasets, and instrument output files that are being used in ongoing related studies. The main processed data supporting the findings are reported in the manuscript.
